# A meal concept designed for older adults – Small, enriched meals including dessert

**DOI:** 10.29219/fnr.v62.1572

**Published:** 2018-10-26

**Authors:** Evelina Höglund, Susanne Ekman, Gunnel Stuhr-Olsson, Christina Lundgren, Berit Albinsson, Michael Signäs, Christina Karlsson, Elisabet Rothenberg, Karin Wendin

**Affiliations:** 1 RISE Research Institutes of Sweden, Agrifood and Bioscience, Göteborg, Sweden; 2 Findus Special Foods, Bjuv, Sweden; 3 Medirest Compass Group AB, Kista, Sweden; 4 ICA Sverige AB, Solna, Sweden; 5 Food and Meal Science, Kristianstad University, Kristianstad, Sweden; 6 Department of Food Science, University of Copenhagen, Copenhagen, Denmark

**Keywords:** older adults, energy, protein, meals, malnutrition, meal concept

## Abstract

**Background:**

The population of older adults is growing and many are at risk of disease-related malnutrition. This is a serious condition which increases the risk for other diseases and distress, human suffering and puts a high load on health care costs. Meal concepts tailored to suit the needs of older adults are required to decrease the incidence of disease-related malnutrition.

**Objective:**

To evaluate sensory perception regarding a concept of small, protein and energy-enriched multi-component meals designed according to the nutritional needs of older adults.

**Design:**

A meal concept of small main courses with complementary desserts and protein and energy-enriched products was evaluated using triangle tests, hedonic evaluation and focus group discussion. Enriched sauces and meals were compared to corresponding commercial products regarding appearance, taste, consistency and overall acceptance.

**Results:**

The concept of a small main course with a complementary dessert was generally perceived as positive by the target group. The acceptance scores for the enriched meals were generally lower than for the commercial meals, mainly owing to the packaging of the enriched meals which required covering the food in sauce. Enriched sauces contained approximately 90% more protein than the commercial sauces. However, protein enrichment affected the sensory properties of the sauces and they were perceived as thicker, creamier and less flavour-intensive.

**Conclusions:**

A concept based on small, protein and energy-enriched meals supplemented with a dessert was considered suitable for increasing the energy and protein intakes of older adults provided that the method of enrichment ensures attractive sensorial properties.

The population of older adults is growing worldwide due to improved living conditions, health care, nutrition and hygiene (1). However, many older adults in the community are frail, a condition whereby a person has decreased physiologic reserve and resistance to stressors resulting from cumulative declines across multiple systems. This results in vulnerability to adverse outcomes (2) and risk for disease-related malnutrition (DRM) (3–5), a condition associated with muscle loss, weakened immune system, morbidity, mortality and poor quality of life (6–8). The aetiology behind DRM is complex but the major cause is acute and chronic disease leading to inflammation, which in turn causes loss of appetite and muscle wasting. Moreover, mastication problems, dry mouth and throat, olfactory dysfunction and social changes may contribute to the risk of malnutrition in old age (7, 9, 10). Disease-related malnutrition places a high economic burden on health care (11, 12) but could be counteracted through the development of meal concepts, including food products, adapted to the changing needs of those affected.

Poor appetite is strongly associated with poor nutritional intake (3, 5, 13). In order to increase nutritional intake, products customised to the needs of older persons have to be designed in a way that encourages consumption of the entire meal (14). Large portions of food may be overwhelming and discourage intake (15). Moreover, food intake is primarily dependent on the volume of food consumed and not its energy content (16, 17). Therefore, a strategy that can be used for older adults who require increased energy and nutrient intakes is to offer frequent, small servings of food with high energy and nutrient density (18). Formulation and processing of such food is crucial since sufficient energy and nutrients need to be provided in a limited meal volume. Older adults need more protein than younger people to maintain physical function. Those who have, or are at risk of, DRM need even higher levels of protein intake to maintain muscle mass (19). In order to achieve high protein concentration in food products, whey proteins are commonly added. These proteins have properties that stimulate muscle protein synthesis (20–22) and can be added to food products such as sauces, gravies and desserts due to functional properties such as foaming, gelling and water-binding capacity (23). However, protein enrichment of food products is not straightforward since the addition of protein affects flavour as well as consistency. Also, olfactory dysfunction is common in older adults which often leads to a reduced ability to appreciate food (24, 25). Therefore, the sensory attributes of meals designed for older adults require careful attention, underlining the importance of taste and flavour (18). Variety among meal components has been reported to promote intakes of larger quantities of food, partly because satiety can be specific to one type of food (5, 26). Therefore, a concept of small, multi-component meals with high energy and nutrient density, such as a protein-enriched main course supplemented by a dessert, may encourage food intake and reduce the risk of DRM. The aim of this study was to evaluate the concept of small protein and energy-enriched meals designed for older adults using sensory analysis. Enriched meals were compared to corresponding commercial meals with regard to appearance, taste and consistency as well as overall acceptance.

## Material and methods

### Study setup

In this study, poor appetite was taken into account and meal size was reduced by 20–30%. The challenge was to reduce the main components of the meals without decreasing the total energy and protein content. In order to compensate for protein reduction in small-sized meals, the sauces and mashed potatoes (when applicable) were protein-enriched. The effects on sensorial properties of protein enrichment of the sauces were evaluated in this study by a discrimination test and hedonic evaluation, while the enrichment of the mashed potatoes was evaluated using only hedonic evaluation.

In order to increase the energy content of the meals, a dessert was added to the main course. The desserts also added variety to the meals which has been suggested to promote food intake. Enriched meals, and their components, were evaluated in a hedonic test concerning appearance, taste, consistency and overall acceptance, and the concept of a small, enriched meal with an attached dessert was discussed in a focus group.

### Samples

Six types of ready-to-eat (RTE) meals were manufactured in a pilot plant at Findus Sverige AB, Bjuv, Sweden. Three of the meals were commercial meals available on the consumer market, and the other three were corresponding meals developed and designed according to the nutritional needs of older adults. These meals were of smaller portion size (70–80% (w) of commercial RTEs), protein-enriched with whey powder (sauces and mashed potatoes) and supplemented with a dessert to increase the total energy content of the meal. The composition of the RTE meals and the separate sauces is shown in Table 1. The samples were packed in black paper/plastic trays covered with a transparent plastic film and the desserts were packed in a side tray. All samples were delivered as frozen goods. The day before testing, sauces were thawed at room temperature (approximately 4 h at 20°C) and then in a refrigerator (approximately 16 h at 4°C). About 1 h before the test, the sauces (2.7–3.1 l) were heated to ≥70°C in steel sauce pans on electric hot plates while being continuously stirred, and approximately 40 ml was then poured into coded plastic cups. The RTE meals were heated prior to testing in identical microwaves ovens (700 W) according to the manufacturer’s instructions. However, the heating time was adjusted in order to reach a minimum temperature of 65°C throughout in each meal.

**Table 1:** Nutritional content of commercial and protein-enriched products

Product		Energy (kcal/100g)	Fat (g/100g)	Carbo-hydrate (g/100g)	Protein (g/100g)
Commercial sauces	Mustard sauce	124	9.1	8.3	2.3
	Cream sauce	180	15	7.6	4.4
	Curry sauce	86	5.1	7.4	2.4
Protein-enriched sauces	Mustard sauce	132	9.6	7.3	4.1
	Cream sauce	163	12	6.6	7.2
	Curry sauce	101	5.9	6.4	5.2
Commercial RTE meals 380–400 g/portion	Fish, mustard sauce* (400 g/portion)	95	3.5	9.5	5.5
	Meat balls, cream sauce** (400 g/portion)	160	8.5	15.0	5.0
	Chicken, curry sauce*** (380 g/portion)	120	3.0	16.0	5.5
Protein-enriched meals 285–310 g/portion	Fish, mustard sauce* (310 g/portion)	101	4.1	9.1	6.3
	Meat balls, cream sauce** (285 g/portion)	151	7.7	14.0	6.0
	Chicken, curry sauce*** (285 g/portion)	121	2.4	16.0	8.2
Concept of protein-enriched meals designed for older adults, including dessert 385 g/portion	Fish, mustard sauce* + Chocolate cake, raspberry cream (Dessert: 75 g/portion)	146	7.5	13.0	5.9
	Meat balls, cream sauce** + Pancake, strawberry sauce (Dessert: 100 g/portion)	156	7.5	16.2	5.5
	Chicken, curry sauce*** + Apple cake, custard (Dessert: 100 g/portion)	174	6.7	20.0	7.8

* including mashed potatoes and peas;

** including mashed potatoes, lingonberries and peas;

*** including rice and broccoli/baby carrots.

### Respondents

The inclusion criteria for the respondents (RPs) were: independent living, aged 75+ years, cognitively intact, able to travel to the location where the tests were conducted and willing to eat/like RTE meals. The goal was to recruit both men and women and reach a slightly higher number of women. Ethical approval is not needed for food focus groups; however, the respondents were informed about the products and the terms for participation, which meant voluntary participation, freedom to leave the test without giving a reason, the right to decline to answer specific questions or an assurance that although their answers were recorded, their participation would not affect their future treatment in the health care system in any way.

### Sensory evaluation

The sensory evaluations of the meals were carried out at the sensory laboratory at RISE Research Institutes of Sweden, equipped according to ISO 8589:2007. Evaluations were carried out in three steps: (1) a triangle test for detection of differences between commercial and enriched sauces, (2) hedonic evaluation to rate the appearance, taste and consistency of commercial and enriched RTE meals, and (3) a focus group discussion to gain a deeper understanding of opinions about RTE meals designed for older adults. The evaluations were carried out over 2 days in the same week. Assessment was made in the following order: mustard sauce, cream sauce and curry sauce (increasing flavour strength).

### Hedonic evaluation

Hedonic evaluation of the RTE meals was conducted on the second test day. The RPs were presented with one meal at a time in the same presentation order: fish with mustard sauce, meat balls with cream sauce and chicken with curry sauce. The commercial meal (with no dessert) was always evaluated before the corresponding enriched meal. The enriched meals included a dessert, which was presented with the main course in order to give the RPs the possibility of giving feedback about the concept of a small main course with dessert. Written information about the name of the meals was provided on the front page of the questionnaire. The test was performed in the sensory laboratory and took 1.5 h. First, the RPs rated the appearance of the meal and its components (mashed potatoes or rice, sauce and protein component) separately before tasting. Finally, all components were considered together to evaluate overall acceptance. The ratings were made on a 9-point hedonic category scale with the ends anchored as ‘dislike extremely’ and ‘like extremely’ (27). Furthermore, the RPs were given the opportunity to comment on each assessment in their own words. Sauces and mashed potatoes were the components that were protein-enriched and both taste and consistency were evaluated for these components. The desserts were evaluated with regard to their total acceptance.

### Triangle test

A triangle test, based on ISO 4120 ‘Sensory analysis, methodology, triangular test’ and described by Lawless and Heymann (28), was conducted in order to determine whether older adults (naïve assessors) could detect any difference between commercial and protein-enriched sauces. Each RP was presented with three coded samples, of which two were identical and one different. The samples were served in random order and the RPs were asked to determine which of the samples was different. Hence, RPs had a one-in-three chance of selecting the correct response. Each RP carried out the triangle tests in triplicate. Thus, a total of 36 assessments were carried out for each sauce. Further, the RPs were asked to indicate the reason for their decision. Still water, mineral water, neutral wafers and apple slices were used to rinse the mouth and palate between samples. A break of 5 min was taken between each test replicate of the same sauce. After completing the three test replicates for one sauce, the RPs had a 10-min pause before the next evaluation.

### Focus group

A focus group discussion was held directly after the hedonic evaluation. A moderator facilitated the discussion which was observed and recorded by an assistant. The focus group was conducted using a semi-structured interview guide and lasted for approximately 1.5 h. The moderator began the focus group by explaining the procedure and asking the RPs to briefly introduce themselves. A semi-structured interview guide was used in order for the focus group to be both structured and at the same time allow follow-up questions and discussion. The frequency and reasons for consuming RTE meals were discussed as well as important attributes of RTE meals. Before starting the next topic for the focus group, the moderator informed the RPs about the meals being evaluated in the hedonic evaluation and the following information was given: the small meals had been protein-enriched in order to meet the increased need for protein among older adults, the enriched meals were of smaller portion size because older adults often have poor appetites, and the small meals contained more energy than the corresponding normal-sized meals. In a subsequent discussion, the RPs were asked to give their opinion of the RTE meals based on the new information they had been given as well as suggest ways for communicating to consumers about meals designed for older adults. Finally, the RPs were asked to give input on improvements of the tested products. The prepared RTE meals were displayed and discussed one at a time.

### Data analysis

Quantitative data from the hedonic evaluation were transferred to Excel (Microsoft Office, 2010) and qualitative data (comments) were summarised in Word (Microsoft Office, 2010). A pairwise comparison (Student’s *t*-test, Microsoft Office Excel, 2010) was performed for each parameter to detect any significant differences (*p* ≤ 0.05) between commercial meals and meals designed for older adults.

Data from the triangle test were calculated according to the following equation (29):

$$X=0.4717z\sqrt{n}+\left[\left(2n+3\right)/6\right]$$

X = minimum number of correct/agreeing judgments (X is an integer or closest higher integer)

n = total number of judgments

if *p* < 0.05 z = 1.64

Qualitative data from the focus group were coded using conventional techniques. First, two researchers independently read through the focus group transcripts and the observation field notes and then they performed an initial coding. This coding captured how the respondents perceived the RTE meals and their opinions about them. The initial codes were then merged and discussed among the researchers, and then clustered according to similarity and common features so as to form categories related to perception and opinions of RTE meals. The use of triangulation in the form of different ‘investigators’ increases the possibility of obtaining credible results (30).

## Results

### Enriched and commercial sauces and meals

The protein-enriched sauces contained approximately 90% more protein than commercial sauces and the protein-enriched mashed potatoes contained approximately 60% more protein than commercial mashed potatoes. The enriched meals (including dessert) contained approximately 50% more energy than commercial meals for two of the varieties, while one of the meals had a similar energy level for both enriched and commercial meals.

A total of 12 RPs (seven women, five men, aged 75–89 years) conducted the triangle test and hedonic evaluation. Two married couples participated, the other RPs lived alone. Seven RPs (four women, three men, all living alone) participated in the focus group discussion.

### Triangle test

The enriched sauces contained approximately 90% more protein (mustard sauce 78%, cream sauce 64%, curry sauce 117%) than corresponding commercial sauces (Table 1). However, protein enrichment affected the sensory properties and there was a significant difference in flavour and/or consistency between the commercial and the enriched sauces (Table 2). The differences were larger for the cream sauce (30 correct answers out of 36 assessments in the triangle tests) than the mustard sauce (22 correct answers) and the curry sauce (18 correct answers). Overall, the commercial sauces were perceived as thinner, less creamy, and more flavour-intensive.

**Table 2:** Results of the triangle test (12 respondents and 3 replicates)

Sauce	Replicate	Number of correct answers	Comments
Mustard	1	8	Commercial: stronger taste (2), saltier, spicier, more sour Enriched: milder taste, milder citrus flavour, stronger taste
	2	6	Commercial: saltier, sharper, stronger taste Enriched: milder (3), thicker
	3	8	Commercial: more flavour/spicier/stronger (4), sweeter Enriched: milder (2), saltier, stronger
	Total	22	
Cream	1	10	Commercial: more pepper (3), spicier, bouillon flavour Enriched: less spicy/pepper (3), milder taste, stronger aftertaste, smoother texture, well thickened
	2	10	Commercial: saltier (2), spicier (2), sweeter, fuller, more flavour Enriched: milder (2), bland, more flavour, creamy, thick
	3	10	Commercial: strong, peppery, sour, mild, thin texture Enriched: milder (3), not so spicy, more sting, salt, thicker
	Total	30	
Curry	1	6	Commercial: less flavour (2), more taste Enriched: less flavour (2), spicier
	2	5	Commercial: stronger after taste, more sharp Enriched: less spicy
	3	7	Commercial: less flavour, sweet, more flavour
	Total	18	Enriched: thick, less flavour

Eighteen correct answers implied a significant difference (*p* ≤ 0.05) between two sauces. Numbers in brackets is number of RPs that gave the same comment.

### Hedonic evaluation

In a hedonic evaluation of the commercial RTE and corresponding enriched meals, the scores were higher for the commercial than for the enriched meals for all three dishes and the differences were significant for the meat ball and chicken dishes (Table 3). For one of the dishes (meat balls), the enriched mashed potatoes had lower scores regarding taste and consistency compared to the commercial meal, but this was not seen for the fish dish. In the case of the separate mustard and curry sauces, there was no difference in taste or consistency between the commercial and enriched sauces. For the cream sauce, however, the commercial sauce appeared to be accepted to a higher extent than the enriched sauce owing to taste and consistency. The triangle test (Table 2) also showed a significant difference between commercial and enriched cream sauces. No differences were found between commercial and enriched meals regarding the rice or the protein component (data not shown). In terms of the overall acceptance, there were no significant differences between the commercial and enriched meals in the case of the fish and meat ball dishes. However, for the chicken dish, the commercial meal had significantly higher acceptance scores than the enriched meal.

**Table 3:** Results of the hedonic evaluation

Hedonic evaluation		Commercial RTE meals	Enriched meals designed for older adults
Appearance	Fish/mustard sauce	4.8	4.7
	Meat balls/cream sauce	6.7*	5.3*
	Chicken/curry sauce	7.5*	5.3*
Mashed potatoes – taste	Fish/mustard sauce	6.2	6.3
	Meat balls/cream sauce	5.9*	4.8*
Mashed potatoes – consistency	Fish/mustard sauce	6.8	6.2
	Meat balls/cream sauce	6.8*	5.0*
Sauce – taste	Fish/mustard sauce	6.8	6.5
	Meat balls/cream sauce	6.4*	5.1*
	Chicken/curry sauce	5.9	5.9
Sauce – consistency	Fish/mustard sauce	7.2	6.3
	Meat balls/cream sauce	6.7*	5.8*
	Chicken/curry sauce	6.3	6.1
Overall acceptance	Fish/mustard sauce	5.8	5.6
	Meat balls/cream sauce	6.1	5.4
	Chicken/curry sauce	7.1*	5.8*

* significant (*p* ≤ 0.05) difference between commercial and enriched meals. RTE, ready-to-eat.

### Focus group discussion

All respondents in the focus group were used to consuming frozen RTE meals. The majority of the female RPs consumed such meals once a month, while the men in general consumed such meals more often, 1–4 times/week. The reasons for consuming frozen RTE meals were almost the same for men and women, lack of time (because of activities) or lack of inspiration or energy. However, in contrast to the women, several men expressed that, due to cultural reasons, they did not have enough cooking knowledge. The RPs felt that frozen RTE meals were attractive meal solutions, easy to store in the freezer (frequent shopping for food is not needed), and quick to cook.

An important attribute of RTE meals was considered to be an appetising and colourful appearance, to which both a sauce and vegetables should contribute. Several women stated that pieces of fish, chicken, or meat should be visible and presented separately, that is, not hidden in a sauce. The taste was considered to be highly important and the meal should be well-seasoned. They also expressed that the provision of a tasty sauce is especially important.

There was a consensus in the group that designing RTE meals, especially for older adults, and making them more nutrient-dense and protein-rich is a very good idea. The majority were positive to the concept of providing a main course supplemented with a dessert. However, opinions differed regarding the addition of a dessert. Some of the RPs thought that the dessert gave an extra value to the meal by adding variety. Conversely, a pair of female RPs found the desserts unnecessary and would rather eat fresh fruit after the main course. When discussing communication to consumers about RTE meals designed for older adults, most RPs found the words ‘protein-rich’ and ‘energising’ both appealing and understandable. However, the word ‘energy-dense’ was perceived as being cryptic. A photo of the food, as well as the food being visible through the packaging, was considered to make the meals more attractive.

## Discussion

The concept of a small, enriched meal with dessert was evaluated in this study. A deeper understanding of the attitudes towards RTE meals and meals designed for older adults was gained through focus group analysis. Olfactory dysfunction, decreased ability to perceive food flavours, and impaired appetite due to ageing and age-related diseases were discussed. These issues have also been reported in the literature since malnutrition in older adults has been linked to social, physiological, and psychological changes (18). Focus group discussions highlighted the necessity of seasoning being adapted to the preferences of older adults and an attractive sauce, regarding both taste and appearance, was considered positive. Appleton (31) reported that supplementation of meals with sauces can increase energy intake in older adults. In the present study, the protein-enriched sauces contained almost twice the amount of protein than the commercial sauces. However, whey protein enrichment of sauces altered the consistency markedly and led to lower flavour intensity. Hence, protein enrichment significantly affected the sensory properties of the sauces due to the physiochemical properties of whey proteins such as their water-binding capacity and emulsifying properties. When designing protein-rich meals, the protein enrichment should not exceed the level whereby sensory properties are significantly affected, instead these effects need to be addressed in the formulation step by using other ingredients that counteract the effects of protein enrichment. In addition, the physiochemical properties of whey proteins may be altered prior to addition by using thermal, chemical, or physical pre-treatments (32–34) enabling improved functionality in food products.

Overall, commercial RTE meals had a higher acceptance score than the corresponding enriched meal for all dishes. One reason for this may be the packaging as the enriched meals were plated so that most of the meal was covered in sauce, which was not appreciated by the RPs. The consistency of the mashed potatoes in the protein-enriched meals was perceived as poor, which could also be linked to food packaging, resulting in the mashed potatoes being covered with sauce. Hence, plating of the food may be the reason for the lower acceptance scores of enriched meals rather than the actual protein enrichment.

Hughes and Bennett (35) reported that poor cooking skills and low motivation to change eating habits among older men may constitute barriers to improving energy intake and appetite. Frailty and disease increase with age and may lead to a decline in the ability to shop and cook independently (2, 36, 37). There is therefore a need for RTE meals with nutritional content adapted to the needs of older adults. The concept of a small main course with a supplementary dessert was generally perceived as positive, but some of the female RPs preferred fresh fruit after the meal rather than a dessert. However, to reduce the risk of DRM, the food products consumed should have a high energy content (38). Hence, an energy-dense dessert could more easily meet the nutritional demands of many older adults than fresh fruit.

## Limitations

The RPs in the focus group were healthier and more alert than the actual target group of the study (frail older adults at risk of malnutrition). This needs to be considered in relation to the analysis of the focus group discussion. It is difficult to recruit a group of frail and sick older adults for a focus group session. The hedonic evaluation was performed with a limited number of RPs. For a more indicative result, a consumer test with a higher number of RPs should be carried out. However, in order to perform all three types of evaluations, a lower number of RPs was chosen. The results of the study should hence be regarded as indications. Similarly, plating of the commercial and protein-enriched meals would exclude the effects of covering most of the meals in sauce. In the present study, only one level of protein enrichment was used. Testing several levels may have indicated an optimal cut-off point for protein enrichment before any significant effects on sensory properties. Also, pre-treatments of whey powder have the possibility to adapt the physiochemical properties of the powder so that the effects on consistency are reduced. No such pre-treatments were included in the present study.

## Conclusion

A concept of small, protein and energy-enriched RTE meals with dessert was evaluated by older adults in terms of sensory properties and attitudes towards this type of meal. Frozen RTE meals were considered to be convenient meal solutions and an attractive sauce was highlighted as important for consumer acceptance. Protein enrichment of sauces, at levels which doubled the protein content, was feasible but altered the consistency markedly and led to lower flavour intensity. Provided that protein enrichment is carried out in a manner that assures attractive sensorial properties, a protein-enriched sauce qualifies as a suitable component in a meal designed for older adults. Plating of the food turned out to be a key factor regarding visibility of meal components (not covered in sauce) and avoidance of poor consistency due to mixing the sauce with other meal components. The meal concept was perceived as successful in increasing energy and protein intakes in older adults; however, the effects of protein enrichment on sensory properties need to be further investigated in order to design tailored foods intended to decrease age-related DRM.

## References

[1] Fund UNP Aging in the twenty-first century. New York: UNFPA and HelpAge International; 2012.

[2] Morley JE, Vellas B, van Kan GA, Anker SD, Bauer JM, Bernabei R, et al. Frailty consensus: a call to action. J Am Med Dir Assoc 2013; 14(6): 392–97. 23764209 10.1016/j.jamda.2013.03.022 PMC4084863

[3] Schlip J, Wijnhoven HAH, DDJ H, Visser M. Early determinants for the development of undernutrition in an older general population: longitudinal aging study Amsterdam. Br J Nutr 2011; 106: 708–17. 21450117 10.1017/S0007114511000717

[4] Shahar D, Shai I, Vardi H, Fraser D. Dietary intake and eating patterns of elderly people in Israel: who is at nutritional risk? Eur J Clin Nutr 2003; 57: 18–25. 12548292 10.1038/sj.ejcn.1601523

[5] van der Meij BS, Wijnhoven HAH, Finlayson GS, Oosten BSH, Visser M. Specific food preferences of older adults with poor appetite. A forced-choise test conducted in various care settings. Appetite 2015; 90: 168–75. 25772198 10.1016/j.appet.2015.03.011

[6] Liu L, Bopp MM, Roberson PK, and Sullivan DH. Undernutrition and risk of mortality in elderly patients within 1 year of hospital discharge. J Gerontol 2002; 57A(11): M741–46. 10.1093/gerona/57.11.m741 12403803

[7] Rasheed S, Woods RT Malnutrition and quality of life in older people: a systematic review and meta-analysis. Aging Res Rev 2012; 12(2): 561–6. 10.1016/j.arr.2012.11.003 23228882

[8] Stratton RJ, Elia M A review of reviews: a new look at the evidence for oral nutritional supplements in clinical practice. Clin Nutr Suppl 2007; 2: 5–23.

[9] Attems J, Walker L, Jellinger KA Olfaction and aging: a mini-review. Gerontology 2015; 61(6): 485–90. 25968962 10.1159/000381619

[10] Mobley AS, Rodriguez-Gil DJ, Imamura F, Greer CA. Aging in the olfactory system. Trends Neurosci 2014; 37(2): 77–84. 24361044 10.1016/j.tins.2013.11.004 PMC3947771

[11] Amaral TF, Matos LC, Tavares MM, Subtil A, Martins R, Nazare M. The economical impact of disease-related malnutrition at hospital admission. Clin Nutr 2007; 26: 778–4. 17936442 10.1016/j.clnu.2007.08.002

[12] Meijers JMM, Halfens R, JG, Wilson L, Schols jMGA. Estimating the costs associated with malnutrition in Dutch nursing homes. Clin Nutr 2012; 31: 65–8. 21880401 10.1016/j.clnu.2011.08.009

[13] Mudge AM, Ross LJ, Young AM, Isenring EA, Banks MD. Helping understand nutritional gaps in the elderly (HUNGER): a prospective study of patient factors associated with inadequate nutritional intake in older medical inpatients. Clin Nutr 2011; 30: 320–5. 21262553 10.1016/j.clnu.2010.12.007

[14] Rothenberg E, Wendin K Texture modification of food for elderly people, in Modifying Food Texture Volume 2: Sensory analysis, Consumer Requirements and Preferences. In Chen J, Rosenthal A, eds. Woodhead Publishing Series in Food Science, Technology and Nutrition: number 284, Cambridge, UK, 2015, 163–85.

[15] Huffman GB Evaluating and treating unintentional weight loss in the elderly. Am Fam Physician 2002; 65(4): 640–50. 11871682

[16] De Castro JM Macronutrient and dietary energy density influences on the intake of free-living humans. Appetite 2006; 46: 1–5. 16171899 10.1016/j.appet.2005.05.005

[17] Goetze O, Steingoetter A, Menne D, van der Voort IR, Kwiatek MA, Boesiger P, et al. The effect of macronutrients on gastric volume responses and gastric emptying in humans: a magnetic resonance imaging study. Am J Physiol Gastrointest Liver Physiol 2007; 292: G11–7. 16935851 10.1152/ajpgi.00498.2005

[18] Nieuwenhuizen WF, Weenen H, Rigby P, Hetherington MM. Older adults and patients in need of nutritional support: review of current treatment options and factors influencing nutritional intake. Clin Nutr 2010; 29: 160–9. 19828215 10.1016/j.clnu.2009.09.003

[19] Bauer J, Biolo G, Cederholm T, Cesari M, Cruz-Jentoft AJ, Morley JE, et al. Evidence-based recommendations for optimal dietary protein intake in older people: a position paper from the PROT-AGE Study Group. JAMDA 2013; 14(8): 542–59. 23867520 10.1016/j.jamda.2013.05.021

[20] de Almeida Marques G, de Sao José JFB, Alves Silva D, da Silva EMM. Whey protein as a substitute for wheat in the development on no added sugar cookies. LWT - Food Sci Technol 2016; 67: 118–26.

[21] Pennings B, Boirie Y, Senden JMG, Gijsen AP, Kuipers H, van Loon LJC. Whey protein stimulates postprandial muscle protein accretion more effectively than do casein and casein hydrolysate in older men. Am J Clin Nutr 2011; 93: 997–1005. 21367943 10.3945/ajcn.110.008102

[22] Yang Y, Breen L, Burd NA, Hector AJ, Churchward-Venne TA, Josse AR, et al. Resistance exercise enhances myofibrillar protein synthesis with graded intakes of whey protein in older men. Br J Nutr 2012; 2012: 1780–8. 10.1017/S0007114511007422 22313809

[23] Chung C, Degner B, McClements DJ Creating novel food textures: modifying rheology of starch granule suspensions by cold-set whey protein gelation. LWT - Food Sci Technol 2013; 54: 336–45.

[24] Doty RL, Kamath V The influences of age on olfaction: a review. Front Psychol 2014; 5(20): 1–20. 24570664 10.3389/fpsyg.2014.00020 PMC3916729

[25] Duffy VB, Backstrand JR, Ferris AM Olfactory dysfunction and related nutritional risk in free-living, elderly women. J Am Dietetic Assoc 1995; 95(8): 879–84. 10.1016/S0002-8223(95)00244-8 7636078

[26] Rolls B Experimental analyses of the effects of variety in a meal on human feeding. Am J Clin Nutr 1985; 42: 932–9. 4061365 10.1093/ajcn/42.5.932

[27] Peryam DR, Girardot NF Advanced taste test method. Food Eng 1952; 24: 58–, 194.

[28] Lawless HT, Heymann H Sensory evaluation of food. ed. F.S.T. Series. 2010, New York: Springer Science + Business Media.

[29] Roessler EB, Pangborn RM, Sidel JL, Stone H. Expanded statistical tables for estimating significance in paired-preference, paired-difference, dou-trio and triangle tests. J Food Sci 1978; 43(3): 940–3.

[30] Lincoln YS, Guba EG Naturalistic inquiry. Beverly Hills, CA: Sage; 1985.

[31] Appleton KM Increases in energy, protein and fat intake following the addition of sauce to an older person’s meal. Appetite 2009; 52: 161–5. 18840490 10.1016/j.appet.2008.09.009

[32] Lam RSH, Nickerson MT The effect of pH and temperature pre-treatments on the physiochemical and emulsifying properties of whey protein isolate. LWT - Food Sci Technol 2015; 60: 427–34.

[33] Oboroceanu D, Wang L, Magner E, Auty MAE. Fibrillization of whey proteins improves foaming capacity and foam stability of low protein concentrations. J Food Eng 2014; 121: 102–11.

[34] Tan MC, Chin NL, Yusof YA, Taip FS, Abdullah J. Improvement of eggless cake structure using ultrasonically treated whey protein. Food Bioprocess Technol 2015; 8(3): 605–14.

[35] Hughes G, Bennett KM, Hetherington MM Old and alone: barriers to healthy eating in older men living on their own. Appetite 2004; 43; 269–76. 15527929 10.1016/j.appet.2004.06.002

[36] Johannesson J, Rothenberg E, Dahlin Ivanoff S, Slinde F. Gender differences in practice, knowledge and attitudes regarding food habits and meal patterns among community dwelling older adults. J Aging Res Clin Pract 2016; 5: 220–8.

[37] Fried LP, Tangen CM, Walston J, Newman AB, Hirsch C, Gottdiener J, et al. Frailty in older adults: evidence for a phenotype. J Gerontol: Med Sci 2001; 56A(3): M146–56. 10.1093/gerona/56.3.m146 11253156

[38] Collins J, Porter H The effect of interventions to prevent and treat malnutrition in patients admitted for rehabilitation: a systematic review with meta-analysis. J Human Nutr Diet 2015; 28: 1–15. 10.1111/jhn.12230 24811842

